# The Circadian Clock Protein BMAL1 Acts as a Metabolic Sensor In Macrophages to Control the Production of Pro IL-1β

**DOI:** 10.3389/fimmu.2021.700431

**Published:** 2021-11-09

**Authors:** George A. Timmons, Richard G. Carroll, James R. O’Siorain, Mariana P. Cervantes-Silva, Lauren E. Fagan, Shannon L. Cox, Eva Palsson-McDermott, David K. Finlay, Emma E. Vincent, Nicholas Jones, Annie M. Curtis

**Affiliations:** ^1^ School of Pharmacy and Biomolecular Sciences and Tissue Engineering Research Group, Royal College of Surgeons in Ireland, Dublin, Ireland; ^2^ School of Biochemistry and Immunology, Trinity Biomedical Science Institute, Trinity College Dublin, Dublin, Ireland; ^3^ Medical Research Council (MRC) Integrative Epidemiology Unit, University of Bristol, Bristol, United Kingdom; ^4^ Translational Health Sciences, Bristol Medical School, University of Bristol, Bristol, United Kingdom; ^5^ Institute of Life Science, Swansea University Medical School, Swansea, United Kingdom

**Keywords:** macrophage inflammation, metabolism, molecular clock, IL-1β, pSTAT3

## Abstract

The transcription factor BMAL1 is a clock protein that generates daily or circadian rhythms in physiological functions including the inflammatory response of macrophages. Intracellular metabolic pathways direct the macrophage inflammatory response, however whether the clock is impacting intracellular metabolism to direct this response is unclear. Specific metabolic reprogramming of macrophages controls the production of the potent pro-inflammatory cytokine IL-1β. We now describe that the macrophage molecular clock, through *Bmal1*, regulates the uptake of glucose, its flux through glycolysis and the Krebs cycle, including the production of the metabolite succinate to drive Il-1β production. We further demonstrate that BMAL1 modulates the level and localisation of the glycolytic enzyme PKM2, which in turn activates STAT3 to further drive *Il-1β* mRNA expression. Overall, this work demonstrates that BMAL1 is a key metabolic sensor in macrophages, and its deficiency leads to a metabolic shift of enhanced glycolysis and mitochondrial respiration, leading to a heightened pro-inflammatory state. These data provide insight into the control of macrophage driven inflammation by the molecular clock, and the potential for time-based therapeutics against a range of chronic inflammatory diseases.

## Introduction

Life on Earth follows a predictable daily rhythm, dictated by the planet’s daily rotation on its axis. This rotation necessitated the evolution of the circadian clock, which allows organisms to anticipate and respond to these predictable environmental changes. Circadian rhythms are oscillations in behaviour and physiology with a 24-hour periodicity and are directed by the central master clock which is located in the suprachiasmatic nucleus (SCN) of the hypothalamus (1). The SCN integrates light signals which synchronize the central clock to the external environment. At the molecular level, these rhythms are generated by a series of interlocking transcription-translation feedback loops (TTFL) centred around the core clock component BMAL1 (2). *Bmal1* is known as the master clock gene as its deletion completely ablates all rhythmic activity throughout the organism (3). BMAL1 and its heterodimerization partner CLOCK bind E-box sites in promoters of clock-controlled genes across the genome. This heterodimer can also induce transcription of the negative arms of the molecular clock which feedback and disrupt the BMAL1-CLOCK heterodimer, thus driving precise 24 hour-rhythms of clock-controlled genes. The SCN clock maintains the synchrony of peripheral clocks, throughout the body *via* rhythmic endocrine and autonomic signalling (4, 5). Peripheral cells, including immune cells of the innate and immune system also express the TTFL to drive circadian rhythms (6–9). Thus, the immune system is highly rhythmic, *via* a network of SCN-driven systemic signals which impact immune cells and endogenous clocks within those immune cells.

Macrophages are central mediators of innate immunity. Many of the key functions of macrophages such as phagocytosis (10–13), cytokine and chemokine production (14–17), and migration (11, 18–20) are under clock control, and display striking differences according to the time of day (21). These oscillations in macrophage function confer protection against a range of pathogens including *L. monocytogenes* (20) and *S. Typhimurium* (22). We previously demonstrated that myeloid BMAL1 protects against lipopolysaccharide (LPS) induced lethality (23), and that BMAL1 maintains an anti-inflammatory environment during experimental autoimmune encephalomyelitis (EAE), a mouse CNS autoimmune disease model (24). BMAL1 also directly regulates the antioxidant transcription factor NRF2 which attenuates inflammation by binding to *il-1β* promoter regions and antioxidant response elements (25). Furthermore, these antioxidant response pathways suppress the hypoxia-inducible factor HIF-1α, a critical regulator of glycolytic metabolism and inflammation, in a ROS-dependent manner. Our lab showed deletion of *Bmal1* led to increased stabilization of HIF-1α and expression of pro-IL-1β, whose promoter is bound by HIF-1α (25, 26). Additionally, BMAL1 has been linked to the regulation of atherosclerosis, although the nature of this regulation is uncertain as data has shown BMAL1 to both attenuate and worsen atherosclerotic pathogenesis (27, 28). Collectively these data implicate macrophage *Bmal1* as an important modulator of innate immunity.

Immunometabolism is a burgeoning area of immunology based on the premise that different intracellular metabolic pathways in immune cells prescribe differential immune phenotype and function (29). For instance, in the mitochondria succinate is a crucial transducer of inflammatory signalling and its partner succinate dehydrogenase (SDH), an enzymatic complex of the electron transport complex (ETC), generates ROS which induces IL-1β in a HIF-1α dependent manner in macrophages (26, 30). Additionally, Krebs cycle metabolites such as citrate (31) and itaconate (32) have shown to be critical immune signalling molecules. Furthermore, glucose uptake and glycolysis have also been implicated to drive macrophage function (33, 34). For instance, the enzyme pyruvate kinase M2 (PKM2) is both a coactivator and target of HIF-1α (35, 36). PKM2 also adopts a monomeric/dimeric configuration upon inflammatory activation which translocates to the nucleus and phosphorylates its targets to promote expression of metabolic and inflammatory gene sets (37). For instance, nuclear PKM2 promotes T cell activation and facilitates M1-like activation of macrophages and expression of IL-1β (38, 39). These range of studies illustrate how metabolic pathways can directly impact upon immune function.

Notably, BMAL1 represses PKM2 transcription and consequently, *Bmal1^-/-^* macrophages display an increased glycolytic metabolism, mediated by increased PKM2 protein expression (40). In the absence of *Bmal1*, the phosphorylation of STAT1 by PKM2 is increased. This led to enhanced STAT1–PD-L1 signalling in the absence of myeloid *Bmal1* and an enhanced sepsis phenotype in a cecal ligation puncture model. BMAL1 overexpression also restrains glycolytic activity to repress M1-like macrophage polarization and alleviate alcoholic liver disease in mice (41). Metabolically influenced STAT3 transcription appears to be clinically important, illustrated by a range of studies showing that PKM2-STAT3 signalling directs Th17 inflammatory signalling to drive EAE and arthritis (42, 43), and IL-1β expression in inflammatory epithelial cells and coronary artery disease macrophages (44, 45).

IL-1β is central to the pathogenesis of many chronic inflammatory diseases such as rheumatoid arthritis, type 2 diabetes, gout, and a vast range of autoimmune conditions (46). The CANTOS trial, which was a randomized control trial of canakinumab, a monoclonal antibody targeting IL-1β, demonstrated that inhibition of IL-1β decreased the rate of recurrent cardiovascular events and that blocking IL-1β could decrease lung cancer incidence and mortality (47, 48). More recently, IL-1β blockade has also revealed potential in terms of ameliorating osteoarthritis whereby patients receiving canakinumab had decreased incident of hip or knee replacement (49). IL-1β is a pivotal cytokine to these and other conditions, therefore, understanding how this cytokine is being regulated is of therapeutic importance.

Overall, this study demonstrates that deletion of BMAL1 releases the brake on cellular metabolism in terms of glucose uptake, glycolysis, and flux through the Krebs cycle, and that these metabolic alterations drive inflammation through the production of IL-1β. We demonstrate two pathways by which BMAL1 represses pro IL-1β, firstly through suppression of SDH enzyme activity and mitochondrial ROS and secondly by the control of PKM2 expression and nuclear localisation, thus preventing pSTAT3-dependent *Il-1β* transcription. This provides a potential mechanism for the rhythmicity in pathology observed in inflammatory conditions such as cardiovascular disease (50), and inflammatory conditions which are aggravated by circadian disruption (51).

## Materials and Methods

### Animals and Reagents

Mice with LoxP sites flanking both sides of exon 4 of *Bmal1* (Bmal1LoxP/LoxP, gifted from the lab of Dr. Christopher A. Bradfield) were crossed with mice containing a lysozyme M activated CRE recombinase (Lyz2Cre, Jackson Labs, #004781) to generate mice with the *Bmal1* gene excised specifically in the myeloid lineage (Bmal1LoxP/LoxPLyz2Cre) i.e. *Bmal1^-/-^*. Mice with a lysozyme M activated CRE recombinase (Lyz2Cre) were used as controls i.e. *Bmal1^+/+^*. Mice used for experiments were both male and female, aged between 8-12 weeks. These mice were bred and maintained in specific pathogen free conditions in the Comparative Medicine Unit (CMU), Trinity College Dublin. All mice were maintained in line with European Union and Irish Health Products Regulatory Authority (HPRA) regulations. Experiments were carried out under HPRA license and with ethical approval from Trinity College Dublin (TCD) bioethics committee and Royal College of Surgeons in Ireland (RCSI) research ethics committee. Lipopolysaccharides from Escherichia coli O55:B5 (L2880), dimethyl malonate (136441), and 2-deoxyglucose (H0887) were purchased from Sigma. Disuccinimidyl suberate (DSS) (21655) was purchased from Thermo Scientific. Stattic (AB120952) was purchased from Abcam. DASA-58 (HY-19330) was purchased from MedChemExpress.

### Culture of Mouse Bone Marrow Derived Macrophages

Mice were euthanized by carbon dioxide inhalation and death was confirmed by cervical dislocation. Hair and tissue was removed from the femurs and tibia and both ends were cut with scissors. A 20G syringe was used to flush bone marrow cells from the bones with cDMEM. The cell suspension was centrifuged at 1500 rpm, the cell pellet was resuspended in red blood cell lysis buffer, warm cDMEM was added after 3 minutes, the cell suspension was passed through a 40μm mesh filter, and the cell suspension was centrifuged at 1500 rpm. The cell pellet was resuspended in cDMEM (supplemented with 10% fetal bovine serum (FBS), 1% penicillin/streptomycin, and 10% L929 cell supernatant which contains macrophage colony stimulating factor), divided between 3 10cm non-treated culture dishes, and differentiated for 6 days in an incubator at 5% CO_2_ and 37°C. Following macrophage differentiation cells were counted and seeded in 6/12/24-well culture dishes. Pretreatments with inhibiting compounds and LPS stimulations are as described by individual figures. Cells were maintained in 25mM glucose as standard, unless otherwise described.

### Seahorse Extracellular Flux Analysis

0.5x10^5^ BMDMs were seeded in a 96-well Seahorse plate. Following treatments cells were washed and incubated with Seahorse XF DMEM Medium pH 7.4 media supplemented with 10 mM glucose, 1 mM sodium pyruvate, and 2 mM glutamine. Seahorse extracellular flux analysis measured oxygen consumption rate (OCR) (pmol/min) and extracellular acidification rate (ECAR) (mpH/min). ECAR was converted into proton efflux rate (PER) (pmolH+/min), a more accurate measure of extracellular acidification, by Seahorse software. Addition of mitochondrial stress test compounds (5 µM oligomycin, 10 µM FCCP, 5 µM rotenone/antimycin) and glycolytic rate assay compounds (5 µM rotenone/antimycin a, 5 mM 2-DG) were made to derive different measures of metabolism.

### Stable Isotope Tracer Analysis

BMDMs were incubated in custom DMEM containing 10 mM U-^13^C_6_ heavy labelled glucose (CLM-1396, Cambridge Isotope Laboratories) and 2 mM unlabelled glutamine and activated with 100 ng/ml LPS for 8 hours. Cells were washed three times with ice-cold saline and lysed in 80% methanol. Cell lysates were dried down using a speed-vacuum concentrator and stored at -80°C. Cellular metabolites were extracted and analysed by gas chromatography-mass spectrometry (GC-MS) using protocols described previously (52, 53). Briefly, metabolite extracts were derived using N-(tert-butyldimethylsilyl)-N-methyltrifluoroacetamide (MTBSTFA). D-myristic acid (750 ng/sample) was added as an internal standard to metabolite extracts, and metabolite abundance was expressed relative to the internal standard. GC/MS analysis was performed using an Agilent 5975C GC/MS equipped with a DB-5MS + DG (30 m × 250 µm × 0.25 µm) capillary column (Agilent J&W, Santa Clara, CA, USA). Metabolite measurements were performed at the Rosalind and Morris Goodman Cancer Research Centre Metabolomics Core Facility supported by the Canada Foundation for Innovation, The Dr. John R. and Clara M. Fraser Memorial Trust, the Terry Fox Foundation (TFF Oncometabolism Team Grand 116128) and McGill University. Mass isotopomer distribution was determined using a custom algorithm developed at McGill University (52).

### Western Immunoblotting

Cells were lysed in Lamelli buffer and samples were separated by SDS polyacrylamide gel electrophoresis. Nitrocellulose or PVDF membranes were probed with antibodies for BMAL1 (14020S, CST), TOM20 (sc-11415, Santa Cruz), β-Actin (4967S, CST) Pro-IL1β (AF-401-NA, CST), Complex II WB Antibody Cocktail (ab110410, Abcam) HIF-1α (14179, CST), GLUT1 (12939, CST), PDHα (3205S, CST), PKM2 (4053, CST), α-Tubulin (3873, CST), Histone H3 (4499, CST), and pSTAT3-Tyr705 (9145, CST). Bands were visualized using an Amersham 680 Imager (GE Healthcare). Densitometry quantification was performed according to the following protocol: http://www.yorku.ca/yisheng/Internal/Protocols/ImageJ.pdf.

### Succinate Injection Assay

0.5x10^5^ BMDMs were seeded in a 96-well Seahorse plate. Following adherence, cells were washed and incubated in a mitochondrial assay solution (MAS) comprised of 220 mM mannitol, 70 mM sucrose, 5 mM MgCl_2_, 10 mM KH_2_PO_4_, 2 mM HEPES, 1 mM EGTA, and 0.2% (w/v) fatty acid free BSA at pH 7.4. Immediately prior to the start of the assay, cells were incubated with MAS supplemented with 4 mM ADP (A2754, Sigma) and 10 µg/ml digitonin (D141, Sigma). OCR measurements of digitonin-permeabilized cells were made before and after the addition of 1.25 mM succinic acid to cells and relative metabolic response to succinate was derived from these measures.

### Flow Cytometry

BMDMs or cells from peritoneal exudate were seeded, stimulated, and detached in PBS. Viability staining was performed using a LIVE/DEAD Near-IR Dead Stain (L10119, Invitrogen). Cells undergoing extracellular staining were incubated with anti-mouse CD16/CD32 (14-0161-85, eBioscience) to block Fc receptors. CD11b (101211, Biolegend) staining was used to gate on macrophage populations from peritoneal exudate. Cells were incubated with MitoTracker Green (M7514, Invitrogen) to measure mitochondrial mass and co-stained with MitoTracker Red (M7512, Invitrogen) to analyse membrane potential. CellROX Deep Red (C10422, Invitrogen) was used to analyse cellular reactive oxygen species. Cells were incubated with the fluorescent glucose analogue 2-NBDG (72987, Sigma) to measure glucose uptake. Cells were analysed using an Attune NxT Flow Cytometer (Thermofisher) and a minimum of 5,000 events were recorded for all samples of a given experiment.

### RT-qPCR

Cells were lysed, RNA was extracted using a PureLink Mini RNA Kit (Invitrogen), and converted to cDNA using a High-Capacity cDNA Reverse Transcription Kit (Applied Biosystems) according to manufacturer instructions. cDNA served as a template for amplification of target genes using SYBR Green mastermix and custom primers. *Rps18*, F: ACTTTTGGGGCCTTCGTGTC, R: GCAAAGGCCCAGAGACTCAT. *Pdha1* F: AAGATGCTTGCCGCTGTATC, R: ATTTGCAAAATTACGGGAAGC. *Pdha2*, F: GTTGTGCCTCGCGTTTCTC, R: CCTCTGAGAGCTGGCTTTTG. *Pdhb*, F: GGAGGGAATTGAATGTGAGG, R: CCACAGTCACGAGATGATTTG. *Sdha*, F: TCGACAGGGGAATGGTTTGG, R: GGACTCCTTCCGAGCTTCTG. *Sdhb*, F: GAGTCGGCCTGCAGTTTCA, R: GGTCCCATCGGTAAATGGCA. *Il-1β*, F: GGAAGCAGCCCTTCATCTTT, R: TGGCAACTGTTCCTGAACTC. *Slc2a1*, F: TATGTGGAGCAACTGTGCGG R: AAGGTTCGGCCTTTGGTCTC. *Pfkfb3*, F: TGGGGCCTTTCAATGTGTGAC, R: ACACTTGTTCTCCGCAAAAACC. *Pgk1*, F: GCTATCTTGGGAGGCGCTAA, R: AAAGGCCATTCCACCACCAA. *Pgm1*, F: GTTGCGAGATGCTGGCTATG, R: CCTGTCAGACCGCCATAGTG. *Eno1*, F: TGCTCTGGTTAGCAAGAAAGTG, R: GTGCCGTCCATCTCGATCAT. PCR reactions were ran on Applied Biosystems 7500 and 7900HT machines. Relative gene expression was determined using a ΔCt calculation using *Rps18* as an internal control.

### Glucose Uptake Assay

Glucose uptake was measured by a 2DG6P-coupled luminescent assay (J1341, Promega). Cells were seeded in a 96-well plate at a density of 0.5x10^5^/well and glucose uptake was assayed according to manufacturer’s instructions.

### Protein Crosslinking

Following LPS stimulation, cells were gently washed and scraped with Dulbecco’s PBS at pH 8. DSS was prepared fresh in DMSO and cells were resuspended in DSS with a final concentration of 500 µM. Crosslinking was performed as cells were incubated for 30 minutes at 37°C. After 30 minutes, the reaction was quenched by adding TRIS HCl pH 7.5 to a final concentration of 25 mM before the cells were lysed in Lamelli buffer under non-reducing conditions and protein was analysed by Western Immunoblot.

### Statistical Analysis

Statistical analysis was performed using Graphpad Prism. All data are representative of at least n=3 independent experiments. Data are presented +/- SEM. Student’s t-tests, multiple student’s t-tests with Holm-Sidak correction, one-way ANOVA with Sidak’s multiple comparisons test, or two-way ANOVA with Tukey’s multiple comparisons test were performed. Significance is reported as *p<0.05, **p<0.01, ***p<0.001, ****p<0.0001. N= numbers refers to the number of animals used to repeat an individual experiment.

## Results

### Mitochondrial Metabolism Is Altered in Macrophages With Deletion of Bmal1

**Figure 1:**
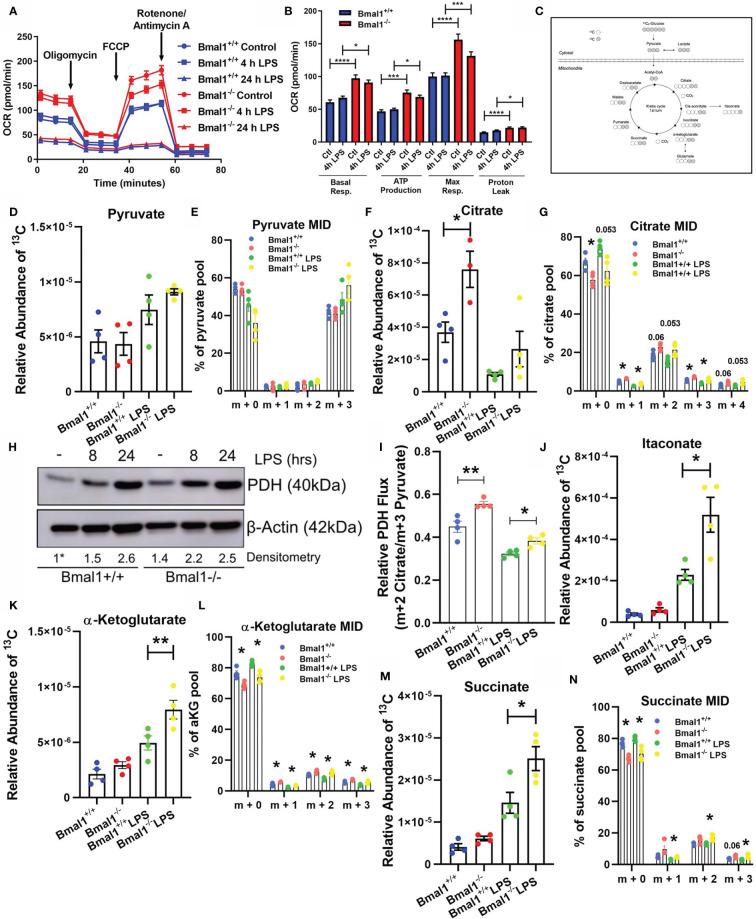
Mitochondrial respiration and Krebs cycle glucose flux is altered in Bmal1^-/-^ macrophages. Bmal1^+/+^ and Bmal1^-/-^ BMDMs were stimulated with LPS (100 ng/ml), subjected to a Seahorse XF mitochondrial stress test. **(A)** OCR of BMDMs was measured, and the metabolic trace illustrates changes in OCR following injection of the mitochondrial stress test compounds oligomycin, FCCP, and rotenone/antimycin a. **(B)** Measures of basal respiration, ATP production, maximal respiration, and proton leak were derived from **(A)**. Assay results are presented +/- SEM and are representative of n=3 independent experiments. BMDMs were isolated, seeded in 10 mM U-13C6 glucose, and stimulated with LPS for 8 hours. BMDMs were lysed and metabolites were quenched and measured *via* GC-MS to trace Krebs cycle flux of labelled glucose. **(C)** A schematic of U-^13^C_6_ glucose-derived carbon Krebs cycle flux and incorporation into metabolic intermediates. Relative abundance of U-^13^C_6_-labelled **(D)** pyruvate, **(F)** citrate, **(J)** itaconate, **(K)** α-Ketoglutarate, and **(M)** succinate, and mass isotopologue distribution (MID) of **(E)** pyruvate, **(G)** citrate, and **(L)** α-Ketoglutarate, and **(N)** succinate were measured. MID values of low abundance isotopologues are excluded for clarity. **(I)** Ratio of m+2 citrate/m+3 pyruvate representative of U-^13^C_6_ glucose-derived carbon flux through pyruvate dehydrogenase. Data presented is n=4 +/- SEM. **(H)** BMDMs were stimulated with LPS and protein expression of PDH was analysed by Western blot using β-Actin as a loading control. Densitometry is relative to the Bmal1^+/+^ control band. This band is indicated by a * symbol. Data presented is representative of n=3 independent experiments. Statistical analysis was performed for Seahorse XF data and U-^13^C_6_ relative abundance values by one-way ANOVA with Sidak’s multiple comparisons test and for MID values by multiple student’s t-tests with Holm-Sidak correction for multiple comparisons (*p < 0.05, **p < 0.01 ***p < 0.001, ****p < 0.0001).

Our previous work described increased ROS in *Bmal1^-/-^* bone marrow derived macrophages (BMDMs) along with localisation of the ROS with mitochondria (25). Therefore, we sought to examine mitochondrial metabolism of BMDMs with deletion of *Bmal1*. BMDMs were prepared from *Bmal1* ^wt/wt^ *Lys-MCre* (i.e. *Bmal1^+/+^*) and *Bmal1* ^LoxP/LoxP^ *Lyz-MCre* (i.e. *Bmal1^-/-^*) mice that have *Bmal1* specifically excised in myeloid lineage cells. BMDMs were stimulated with lipopolysaccharide (LPS, 100 ng/ml) and subjected to a mitochondrial stress test. *Bmal1^-/-^* BMDMs demonstrated higher oxygen consumption rate (OCR) basally and following 4 hours of LPS stimulation but no differences were evident after 24 hours of LPS, in line with the typical kinetics of Warburg metabolism in macrophages (26, 54) (**Figure 1A**). Overall, *Bmal1^-/-^* BMDMs displayed higher mitochondrial metabolism *versus Bmal1^+/+^* in terms of basal respiration, ATP production, maximal respiration, and proton leak basally and following 4 hours of LPS stimulation (**Figure 1B**). However, no effects on mitochondrial mass in terms of protein expression of the mitochondrial marker TOM20 (**Figure S1A**) or fluorescent expression of Mitotracker Green (**Figure S1B**) were observed with *Bmal1* deletion. Mitochondrial membrane potential (**Figure S1C**) was largely unchanged between genotypes apart from a slight but non-significant increase in *Bmal1^-/-^* cells after 1 hour of LPS stimulation.

Given the observed alterations in oxidative metabolism in *Bmal1^-/-^* BMDMs, we next sought to probe the effect of this deletion on Krebs cycle metabolites. The Krebs cycle is a critical pathway generating biosynthetic intermediates and reduced NAD, essential in contributing towards cellular biomass and energetic functions of the mitochondria. We incubated BMDMs with uniformly labelled (U-^13^C_6_) glucose in the presence or absence of LPS for 8 hours, allowing us to detect the fate of glucose-derived carbons through central metabolic pathways (**Figure 1C**).

Initially, we found no significant difference in the levels of ^13^C incorporation into pyruvate between *Bmal1* ^+/+^ and *Bmal1* ^-/-^ macrophages (**Figures 1D, E** **)**. However, when we analysed the Krebs cycle metabolite citrate, we discovered elevated levels of ^13^C incorporation in *Bmal1* ^-/-^ macrophages (**Figure 1F** **)**. Here, the proportion of the m+2 isotopologue of citrate was higher in Bmal1^-/-^ in comparison to Bmal1^+/+^, indicating increased cycling into Krebs cycle intermediates (**Figure 1G**). Given the increased contribution of ^13^C to the citrate pool of *Bmal1^-/-^* macrophages despite no difference in the relative contribution of ^13^C to their pyruvate pool, we next examined the expression of the pyruvate dehydrogenase complex (PDH). RNA expression of individual PDH subunits did not differ between genotypes (**Figures S1D–F**), however protein levels of PDH were increased in *Bmal1* ^-/-^ macrophages during basal conditions and after a short LPS stimulation (**Figure 1H** and **Figures S1G, H**). In order to investigate the flux of pyruvate-derived carbons through PDH (55), we analysed the ratio of total m+2 citrate (i.e. citrate originating *via* PDH metabolism) to total m+3 pyruvate (i.e. total pyruvate generated through glycolysis). Here, relative flux of pyruvate through PDH was higher in *Bmal1^-/-^* cells (**Figure 1I**).

Furthermore, analysis of the downstream Krebs cycle intermediates itaconate, alpha-ketoglutarate and succinate demonstrated significantly increased ^13^C incorporation in LPS activated *Bmal1^-/-^* macrophages *versus Bmal1^+/+^* (**Figures 1J–N**). Downstream of the Krebs cycle break at succinate, the Krebs metabolites fumarate and malate showed an increased trend of ^13^C incorporation however this did not reach significance (**Figures S1I, J**). Overall, these data demonstrate that in the absence of macrophage *Bmal1* an increased flux of glucose-derived carbons into Krebs cycle metabolites is observed, both basally and under LPS activating conditions.

### Mitochondrial Dysfunction Is Driving Heightened Pro IL-1β Expression in Macrophages With Deletion Of Bmal1

**Figure 2:**
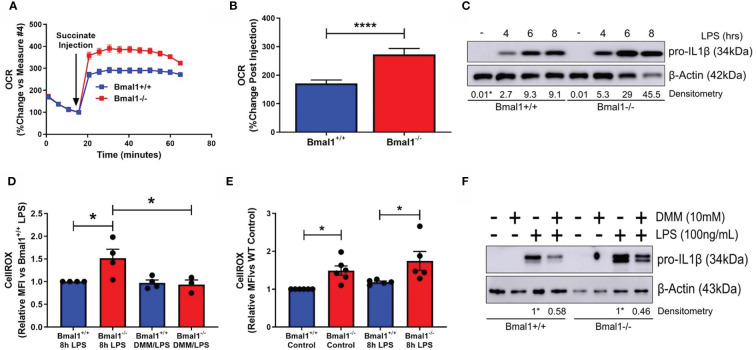
SDH-derived ROS are driving increased inflammation in macrophages with deletion of Bmal1. Bmal1^+/+^ and Bmal1^-/-^ BMDMs were incubated in an isosmotic, ADP-supplemented mitochondrial assay solution and permeabilized with digitonin. **(A)** Seahorse XF analysis was used to analyse the change in OCR in response to injection of succinate (1.25 mM). **(B)** Response to succinate was measured in terms of %change in OCR directly following injection of succinate. Assay results are presented +/- SEM and are representative of n=3 independent experiments. BMDMs were stimulated with LPS (100 ng/ml) and **(C)** stained with CellROX to measure levels of reactive oxygen species by flow cytometry or **(D)** lysed for analysis of pro IL-1β protein expression by Western blot using β-Actin as a loading control. **(E)** Reactive oxygen species and **(F)** pro IL-1β protein expression were measured following pretreatment with DMM before stimulation with LPS. Western immunoblot data presented is representative of n=3 independent experiments. Pro IL-1β time course densitometry is relative to the Bmal1^+/+^ control band. Densitometry for Bmal1^+/+^ and Bmal1^-/^DMM/LPS bands is relative to their LPS bands. These bands are indicated by * symbols. Flow cytometry data presented is at least n=3 independent experiments +/- SEM with each data point representative of at least 5,000 events from one sample. Statistical analysis was performed for Seahorse XF data by unpaired student’s t test and for flow cytometry data by two-way ANOVA with Tukey’s multiple comparisons test (*p < 0.05, ****p < 0.0001).

We next sought to investigate whether the increased succinate observed with *Bmal1* deletion and its oxidation may explain the increased pro IL-1β production as we had previously observed (25). In order to investigate SDH enzymatic activity, we permeabilized BMDMs with digitonin. BMDMs received a single injection of succinate (1.25mM) to fuel SDH and elicit an increase in oxygen consumption rate, providing a direct measure of SDH enzymatic activity (**Figure 2A**). *Bmal1^-/-^* BMDMs demonstrated an increased OCR response *versus Bmal1^+/+^* BMDMs following succinate injection (**Figure 2B**), indicating increased SDH activity. RNA and protein expression of SDHa and SDHb subunits were similar between *Bmal1^+/+^* and *Bmal1^-/-^* cells (**Figures S2A–C**) signifying that *Bmal1* deletion in macrophages led to higher enzymatic activity of the complex and not protein abundance.

We next characterised pro-IL-1β production in the absence of macrophage *Bmal1*. As we have previously reported (25), we observed higher levels of pro-IL-1β protein in response to short LPS stimulations in *Bmal1^-/-^* macrophages (**Figure 2C** and **Figures S2E, F**). *Il1b* mRNA expression was higher in *Bmal1^-/-^* macrophages at 4 hours post LPS stimulation (**Figure S2D**). In agreement with our previous findings, we also observed higher basal and LPS-induced ROS in the absence of *Bmal1* in BMDMs as measured by CellROX (**Figure 2D**). Basal ROS levels in peritoneal macrophages were also higher in those cells with deletion of *Bmal1* (**Figure S2G**). Importantly, using dimethyl malonate (DMM) to directly inhibit SDH, we attenuated the increased basal ROS levels (**Figure 2E** **)** and increased LPS-induced pro-IL-1β protein expression (**Figure 2F** and **Figures S2H, I**) of *Bmal1^-/-^* cells towards *Bmal1^+/+^* levels. Therefore, in macrophages lacking *Bmal1*, greater SDH enzymatic activity, coupled with higher succinate, causes increased ROS leading to enhanced pro IL-1β protein expression.

### HIF-1α Regulated Glucose Metabolism Fuels Increased IL-1β Expression in Macrophages With Deletion Of Bmal1

**Figure 3:**
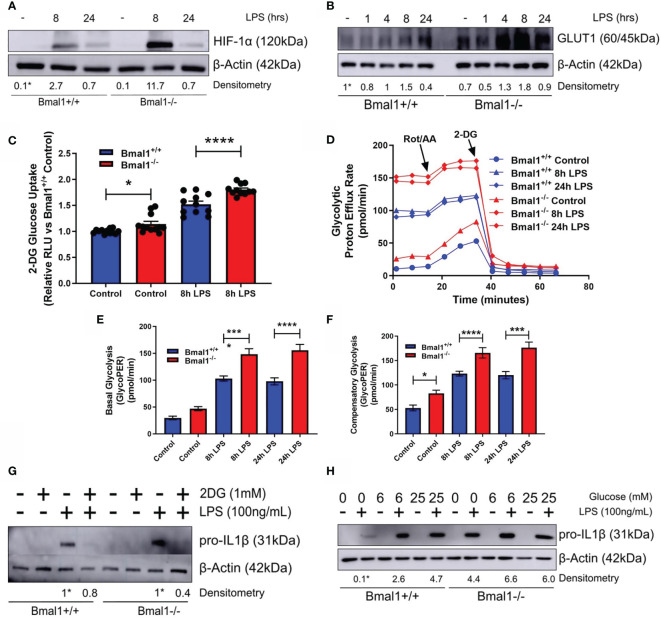
Bmal1^-/-^ macrophages display heightened glucose metabolism which is driving increased pro IL-1β expression. Bmal1^+/+^ and Bmal1^-/-^ BMDMs were stimulated with LPS (100 ng/ml) and lysed for analysis of **(A)** HIF-1α and **(B)** GLUT1 protein expression by Western blot using β-Actin as a loading control. **(G)** Pro IL-1β protein expression was measured following pretreatment with 2DG (1 mM) before stimulation with LPS. Expression of **(H)** pro IL-1β was measured following incubation of BMDMs with different concentrations of glucose before LPS stimulation. HIF-1α time course and glucose/2DG IL-1β Western immunoblot data presented is representative of n=3 independent experiments. GLUT1 time course Western immunoblot data presented is representative of n=2 independent experiments. Time course densitometry is relative to the Bmal1^+/+^ control band. Densitometry for Bmal1^+/+^ and Bmal1^-/^2DG/LPS bands is relative to their LPS bands. Glucose/LPS IL-1β densitometry is relative to the 0 mM glucose/LPS Bmal1^+/+^ band. These bands are indicated by * symbols. **(C)** BMDMs were stimulated with LPS and glucose uptake was analysed *via* luminescent 2-DG uptake assay. Data presented is n=3 +/- SEM. Following LPS stimulation, BMDMs were subjected to a Seahorse XF glycolytic rate assay. ECAR of BMDMs was measured and converted into PER. **(D)** Metabolic trace illustrating changes in PER following injection of the glycolytic rate assay compounds rotenone/antimycin a and 2-DG. Measures of **(E)** basal glycolysis and **(F)** compensatory glycolysis were derived from **(D)**. Assay results are presented +/- SEM and are representative of n=3 independent experiments. Statistical analysis was performed for glucose uptake and Seahorse XF data by one-way ANOVA with Sidak’s multiple comparisons test (*p < 0.05, ***p < 0.001, ****p < 0.0001).

As succinate and SDH drive *Il1b* mRNA expression in a HIF-1α dependent manner (26, 39) and HIF-1α controls glucose metabolism (56) and myeloid cell inflammation (57), we next characterized the expression of HIF-1α in response to LPS. *Bmal1^-/-^* macrophages demonstrated greater HIF-1α protein expression *versus Bmal1^+/+^* after 8 hours of LPS stimulation (**Figure 3A** and **Figures S3A, B**). We also analysed the differential expression of HIF-1α targets between genotypes. GLUT1 protein expression was increased in *Bmal1^-/-^* macrophages following short LPS stimulations (**Figure 3B** and **Figure S3C**). An increase in *Slc2a1* mRNA, the gene which encodes GLUT1, was also observed 8 hours after LPS stimulation (**Figure S3D**) Higher glucose uptake in *Bmal1^-/-^* BMDMs was observed by a 2-DG uptake assay (**Figure 3C**). 2-NBDG uptake was not different between genotypes (**Figure S3E** **)**. This observation is in line with previous findings which reports a disconnect between cellular glucose uptake capacity and 2-NBDG labelling (58). We also found mRNA expression of HIF-1α regulated glycolytic pathway enzymes, including *Pfkfb3*, *Pgk1*, *Pgm1*, and *Eno1* to be increased in *Bmal1^-/-^* cells *versus Bmal1^+/+^* after LPS stimulation (**Figures S3F-I**). *Bmal1^-/-^* BMDMs demonstrated a higher rate of glycolysis basally and after LPS stimulation (**Figure 3D**). Measures of basal (**Figure 3E**) and compensatory (**Figure 3F**) glycolysis derived from the Seahorse XF trace in **Figure 3D** illustrate this higher glycolytic activity pre and post LPS stimulation. Similarly, higher levels of lactate, the end product of anaerobic glycolysis, after 24 hours of LPS stimulation in *Bmal1^-/-^* BMDMs was determined by a colorimetric lactate assay (**Figure S3J**).

Next, we utilized the glucose analogue 2DG which inhibits glycolysis. *Bmal1^+/+^* and *Bmal1^-/-^* BMDMs were incubated with 2DG prior to LPS stimulation and 2DG was able to attenuate the higher expression of IL-1β back to control levels with *Bmal1* deletion (**Figure 3G** and **Figures S3K, L**). We also observed that increased glucose supplementation of the media led to increased expression of pro-IL-1β (**Figure 3H** and **Figures S3M, N**), which was higher with *Bmal1* deletion. Therefore, upon deletion of *Bmal1* in BMDMs, HIF-1α regulated glucose metabolism is upregulated which is known to facilitate increased glucose uptake, glycolysis, Krebs cycle metabolism, succinate, and SDH activity. Collectively, these metabolic activities typically lead to increased ROS and HIF-1α stabilization and further the production of pro-IL-1β. Overall, our results indicate that BMAL1 is a metabolic sensor governing inflammation by way of HIF-1α regulated glucose metabolism.

### Nuclear PKM2 and STAT3 Phosphorylation Drive Increased Expression of IL-1β in *Bmal1^-/-^* Macrophages

**Figure 4:**
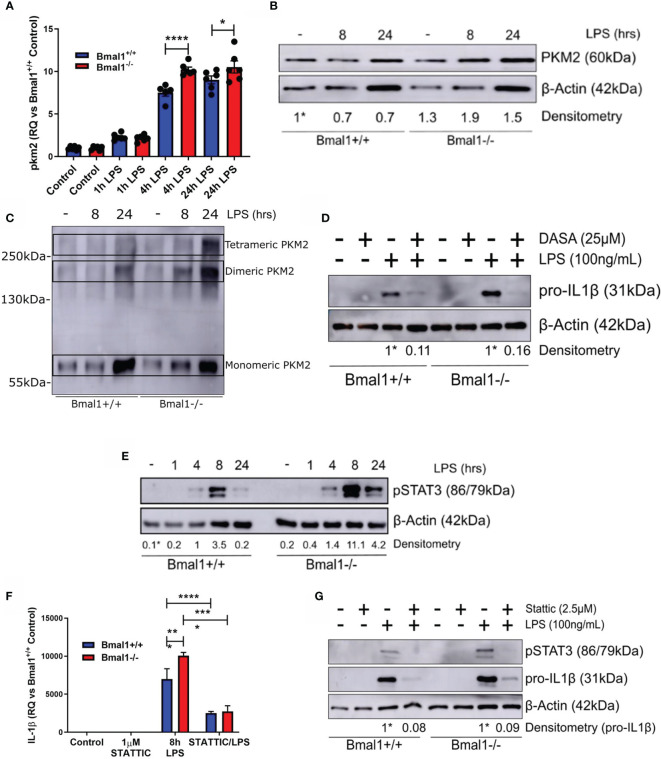
Phosphorylation of PKM2 and STAT3 promote heightened IL-1β expression in macrophages with deletion of Bmal1. WT and *Bmal1* ^-/-^ BMDMs were stimulated with LPS (100 ng/ml), RNA was isolated, and gene expression of **(A)** PKM2 and **(F)** IL-1β were analysed by RT-qPCR. Samples were normalized to their expression of the housekeeping gene 18S. Data presented is n=3 +/- SEM. Statistical analysis was performed by one-way ANOVA with Sidak’s multiple comparisons test (*p < 0.05, ****p < 0.0001). Protein expression of **(B)** PKM2 and **(E)** pSTAT3 was analysed by Western blot using β-Actin as a loading control. **(C)** PKM2 tetramers, dimers, and monomers were resolved by crosslinking samples after LPS stimulation before Western blot analysis. Pro IL-1β protein expression was measured following pretreatment with **(D)** DASA-58 (25 µM) or **(G)** STATTIC (2.5 µM) before stimulation with LPS for 8 hours. Western immunoblot data presented is representative of n=3 independent experiments. PKM2 and pSTAT3 time course densitometry is relative to the Bmal1^+/+^ control band. Densitometry for Bmal1^+/+^ and Bmal1^-/-^ DASA/LPS bands is relative to their LPS bands. These bands are indicated by * symbols. *p < 0.05, **p < 0.01 ***p < 0.001, ****p < 0.0001.

Out of all the HIF-1α dependent glycolytic enzymes analysed, PKM2 displayed the most significant differences in mRNA expression with LPS induction between genotypes (**Figure 4A**). Therefore, we sought to further analyse PKM2 expression in *Bmal1^-/-^* BMDMs and determine if it was contributing to the observed heightened expression of IL-1β. PKM2 protein levels were increased in *Bmal1^-/-^* BMDMs both under basal and LPS stimulated conditions (**Figure 4B** and **Figures S4A, B**). During inflammation, PKM2 is phosphorylated at Tyr-105 which inhibits the formation of its enzymatically active tetrameric form and promotes the formation of monomeric/dimeric PKM2 which localize to the nucleus. Therefore, to investigate the consequences of increased PKM2 phosphorylation in *Bmal1^-/-^* BMDMs, we performed protein crosslinking using DSS and analysed protein expression of PKM2 under non-reducing conditions. Collectively, these experiments demonstrated that expression of nuclear PKM2 monomers and dimers are highly upregulated in response to LPS stimulation (**Figure 4C**, **Figures S4C, D**). Subsequently, we also investigated the effect of pharmacological manipulation of PKM2 configuration using the PKM2 activator DASA-58 which promotes formation of PKM2 tetramers. Pre-treatment of BMDMs with DASA-58 abrogated the heightened pro-IL-1β levels observed in *Bmal1^-/-^* cells (**Figure 4D**, **Figures S4E, F**). Overall, these results indicate increased abundance of nuclear PKM2 in macrophages with *Bmal1* deletion and illustrate that its increased expression is driving the production of the pro-inflammatory cytokine IL-1β.

Notably, the nuclear kinase activity of PKM2 has emerged as an important regulator of immune cell inflammatory activity through phosphorylation of STAT3. Therefore, we next sought to investigate whether the heightened expression of nuclear PKM2 in *Bmal1^-/-^* led to increased STAT3 phosphorylation and increased levels of pro-IL-1β. Nuclear PKM2 can phosphorylate STAT3 at Tyr-705, promoting its activation and downstream inflammatory signalling. Thus, we characterized the expression of pSTAT3 (Tyr-705) in WT and *Bmal1^-/-^* BMDMs in response to LPS (**Figure 4E** and **Figures S5A, B**). pSTAT3 was induced by LPS and its heightened expression in *Bmal1^-/-^* cells peaked after 8 hours of LPS stimulation. Additionally, 2DG pre-treatment attenuated the increased LPS-induced pSTAT3 expression in both genotypes (**Figures S5C, D**). This indicated that glucose metabolism was mediating the LPS-induced pSTAT3 response. Next, to directly investigate whether the enhanced STAT3 Tyr705 phosphorylation in *Bmal1^-/-^* BMDMs leads to increased pro-IL-1β, we utilized the STAT3 inhibitor STATTIC, which prevents the phosphorylation and activation of STAT3 at Tyr-705 and Ser-727 (59). Pre-treatment of BMDMs with STATTIC had no effect on production of IL-6 (**Figure S5E**) or TNFα (**Figure S5F**). However, STATTIC effectively abrogated the increased pSTAT3 (Tyr-705) and IL-1β expression of *Bmal1^-/-^* BMDMs in terms of both RNA (**Figure 4F**) and protein (**Figure 4G** and **Figures S5G, H**) expression. By demonstrating the profound ability of STATTIC to inhibit the heightened expression of IL-1β in *Bmal1^-/-^* cells, in addition to illustrating BMAL1 control of the localization of nuclear PKM2 with STAT3, we therefore identify a novel role for the circadian clock in controlling macrophage immunometabolism.

## Discussion

In this paper, we demonstrate that the molecular circadian clock component BMAL1 impacts on macrophage metabolism to control the expression of the archetypal pro-inflammatory cytokine IL-1β. Our lab has previously shown that BMAL1 controls IL-1β transcription in macrophages (25) whereas others have identified a BMAL1 effect on metabolic pathways impacting immune function (40, 60). Our data further advances the concept that the core circadian clock protein BMAL1 is a key controller of macrophage immunometabolism. Specifically, we uncover two distinct metabolic pathways affecting IL-1β production under the control of BMAL1. We demonstrate that in the absence of *Bmal1*, increased succinate metabolism and elevated PKM2-pSTAT3 pathway activity drive increased pro-IL-1β expression. These mechanisms reveal how *Bmal1* acts as a metabolic sensor in immune cells linking circadian controlled metabolism to the inflammatory response.

BMAL1 has previously been shown to control mitochondrial respiration in muscle (61, 62) and liver (63, 64), where the PERIOD molecular clock proteins have been specifically implicated to regulate the differential usage of pyruvate and fatty acids by the mitochondria throughout the day by driving rhythmic expression of electron transport chain complexes and mitochondrial enzymes (65). Our observation of heightened oxygen consumption in macrophages lacking *Bmal1* is particularly relevant given that mitochondria are now considered as essential nodes in the immune response. The Krebs cycle is of particular importance to macrophage immunometabolism (66). We show that *Bmal1* governs macrophage inflammation by regulating Krebs cycle glucose flux. Flux of glucose-derived carbons through PDH is increased upon deletion of *Bmal1*, providing a potential route for their increased abundance of Krebs cycle intermediates. Dynamic remodelling of the Krebs cycle occurs in macrophages during an inflammatory response and PDH has been implicated as a crucial node linking metabolic changes to inflammatory output (55). HIF-1α stabilization can downregulate PDH expression and result in decreased Krebs cycle flux and oxygen consumption in hypoxic and cancer cells. However, in the context of an LPS stimulated macrophage, flux of glucose derived carbons through PDH is sustained which facilitates the increased synthesis of Krebs cycle intermediates which have important inflammatory functions (55). In line with this data, our results show increased glucose-derived carbon incorporation into metabolites throughout the Krebs cycle of macrophages upon deletion of *Bmal1*. For instance, citrate abundance is significantly greater in *Bmal1^-/-^* macrophages, which corresponds to the increased flux of glucose derived carbons through PDH under basal conditions. Following LPS stimulation we observe a decrease in citrate abundance which may result from citrate being used as a carbon source for lipogenesis, reactive species production, and histone modifications upon inflammatory activation (31). Downstream of citrate, abundance of itaconate, α-ketoglutarate, and succinate are increased following LPS stimulation and higher in *Bmal1^-/-^* macrophages, therefore breakdown of citrate is increased upon macrophage activation and modulated by *Bmal1*. The immunomodulatory metabolite itaconate has recently emerged as a critical determinant of macrophage inflammation, its activity resolving inflammation through regulation of Nrf2 (67) and SDH (68). The increased itaconate abundance evident in *Bmal1^-/-^* macrophages may be reflective of an attempt to quench the higher pro-inflammatory signature in these cells. Collectively, these data suggest that BMAL1 fine tunes Krebs cycle glucose flux in macrophages to impact on the inflammatory response.

Intriguingly, we observed increased glucose-derived succinate levels in macrophages lacking *Bmal1*. Succinate is actively involved in the transduction of inflammation through 1) its action at succinate receptors, 2) its post-translational succinylating activity, and 3) by its accumulation facilitating increased SDH-derived ROS production which inhibit HIF-1α prolyl hydroxylases allowing for HIF-1α stabilization (69). We build on previous work by our lab of heightened HIF-1α stabilisation and increased ROS levels in *Bmal1* ^-/-^ macrophages (25). We show that higher SDH activity is the specific source of ROS which is mitochondrial in origin and leads to increased IL-1β. The pathway connecting succinate metabolism to the expression of IL-1β is well characterized (26, 30), with our data showing the regulatory influence of the molecular clock on this crucial inflammatory pathway. Recent findings by Alexander et al. (60) support this pathway. BMAL1 acts as an environmental sensor to fine-tune HIF-1α activity and bioenergetics in terms of fuel utilization in muscle and liver (61, 62, 64). Our data now extends this role of BMAL1 into an immunometabolic setting in its control of IL-1β production. The relationship of succinate metabolism and HIF-1α is a key link between oxidative metabolism and glycolysis, oxidation of succinate facilitating HIF-1α stabilization and a switch to a glycolytic profile (30). We show that BMAL1 can both impact upon the production of IL-1β and glycolytic phenotype through SDH.

Similarly to Alexander et al. (60) we found glucose uptake, glycolytic pathway activity, and lactate levels to be increased with deletion of *Bmal1* all of which are dependent on HIF-1α. GLUT1-mediated glucose uptake in macrophages is upregulated upon LPS stimulation (33) and is required for M1-like polarization (70). Expression of the GLUT1 glucose transporter is increased in *Bmal1^-/-^* macrophages which facilitates their heightened glucose uptake. The increased expression of GLUT1 in *Bmal1^-/-^* macrophages basally is likely due to higher basal ROS (71) whereas upon LPS stimulation the increase is likely due to the increased stabilization of HIF-1α (72). The heightened expression of GLUT1 in *Bmal1^-/-^* macrophages may be fuelling their increased glycolysis and Krebs cycle glucose flux. Glucose can drive IL-1β expression in macrophages through NFκB activation (73) and glucose-driven IL-1β can positively feedback to further enhance glucose metabolism and inflammation (74). We demonstrate an increased responsiveness of IL-1β expression to alterations in glucose metabolism and concentration in *Bmal1^-/-^* macrophages. Given the role IL-1β plays in perpetuating chronic inflammation in metabolic disorders and that circadian disruption further enhances susceptibility to these disorders (75), our findings may identify BMAL1 as a critical regulator of this glucose metabolism-inflammation feedback loop. Under steady state deletion of *Bmal1* causes circadian disruption and a low level of basal inflammation and metabolism dysfunction. Upon inflammatory stimulation, *Bmal1^-/-^* macrophages are primed to further increase their metabolism, promoting additional inflammation, and propagation of this feedback loop. Therefore, we propose that BMAL1 acts as a critical node in the regulation of glucose driven inflammation.

While the differential expression of glycolytic genes was not largely affected with *Bmal1* deletion, those which showed higher induction with *Bmal1* deletion are targets of HIF-1α. *Pkm2* was one of these genes which had enhanced mRNA and protein expression in *Bmal1^-/-^* macrophages in line with previous findings by Deng. et al. (40). PKM2 is an intensely studied glycolytic enzyme in the field of immunometabolism due to its ability to also moonlight in the nucleus where it acts as a transcriptional regulator. For instance, nuclear PKM2 forms a complex with HIF-1α in macrophages to promote expression of IL-1β (39). Additionally, neutrophils isolated from patients with severe COVID-19 were shown to have increased expression of nuclear PKM2 (76), further implicating PKM2 in the transduction of pathogenic inflammatory signalling. We identified enhanced formation of PKM2 monomers and dimers with *Bmal1* deletion which can readily enter the nucleus. Nuclear translocation of PKM2 mediates the induction of glycolysis after LPS stimulation through its promotion of HIF-1α transcription (39, 40), a mechanism which may therefore be driving the heightened glycolysis we observe in *Bmal1^-/-^* macrophages. Finally, we found that activation of PKM2 using DASA-58, which forces cytosolic localization, abrogated the higher expression of pro-IL-1β in *Bmal1^-/-^* macrophages.

We next sought to understand how the increased nuclear PKM2 observed with *Bmal1* deletion was impacting on IL-1β production. Here, we looked to the STAT transcription factor family as it is heavily involved in inflammatory signalling and is a target of PKM2 kinase activity. Previous evidence had shown that mice lacking myeloid *Bmal1* displayed heightened PKM2 mediated STAT1 phosphorylation in macrophages, which led to T cell exhaustion and an increase in sepsis (40) and that PKM2 phosphorylates STAT1 to activate macrophages in a model of arthritis (77). In terms of STAT3, its phosphorylation by nuclear PKM2 has recently been implicated to mediate the pathogenic phenotype of coronary artery disease macrophages (45), inflammatory lung epithelial cells (44), EAE Th17 cells (43), and CD4+ T cells in a model of arthritis (42). Therefore, the PKM2-STAT3 pathway is emerging as a common pathway across a range of chronic inflammatory conditions.

We observed increased STAT3 phosphorylation at Tyr-705 in *Bmal1* ^-/-^ macrophages. Importantly, using the pSTAT3 inhibitor STATTIC we could bring the increased IL-1β expression of *Bmal1^-/-^* macrophages down to WT levels, confirming the contribution of PKM2/STAT3 axis on BMAL1 controlled IL-1β. These data are particularly relevant given the involvement of the macrophage molecular clock in diseases which also involve the PKM2-STAT3 pathway. For instance, myeloid *Bmal1* deletion in mice has been purported to worsen atherosclerosis driven by ApoE knockout by enhancing vascular inflammation (27). However, it is important to note that myeloid *Bmal1* deletion has been demonstrated as protective against atherogenesis in LDLR-knockout driven hyperlipidemic mice (28). Loss of myeloid *Bmal1* also exacerbates EAE and worsens the pro-inflammatory features of lung inflammation, both of which are also settings that involve PKM2 or STAT3 activity (38, 44).

Our findings demonstrate a dual effect of *Bmal1* deletion on succinate metabolism and PKM2-STAT3 pathway activity to promote increased expression of pro-IL1β, which also appear to be physiologically linked. Succinate can promote HIF-1α stabilization whose target PKM2 can enter the nucleus to phosphorylate STAT3 (26, 36, 45). Additionally, increased levels of succinate are known to generate mitochondrial ROS in inflammatory macrophages and mitochondrial ROS promote phosphorylation of STAT3 *via* localization of PKM2 dimers to the nucleus (30, 45). This suggests mitochondrial ROS as the mechanism by which BMAL1 links these metabolic pathways to synergistically promote inflammation. In a general manner, we also illustrate the regulatory impact of the clock across the immunometabolic landscape to provide rationale for future studies in the area of circadian immunometabolism.

However, it is necessary to note the use of only one model of genetic clock disruption as a limitation to our study. Future studies should assess the effect of disruption to other clock components on the specific pathways investigated here, given the well characterized effects of the clock on immunometabolism (78, 79). The molecular clock is comprised of many linked components, therefore the effects we see due to deletion of Bmal1 may in fact occur as a result of downstream effects on other clock proteins. It is also important that our specific findings are translated into *ex-vivo* and *in-vivo* models given that the effect of *Bmal1* deletion on immunometabolism has been previously demonstrated in such models by Deng et al. and Alexander et al., for example (40, 60). The use of environmental models of clock disruption (jet-lag, constant light dark models etc.) to further investigate our findings would also help to frame our results in terms of wider circadian rhythmicity. Nonetheless, our findings clearly illustrate the impact of Bmal1 on immunometabolism at the molecular and cellular level. Additionally, while these results have been generated in an unsynchronized *in-vitro* system, Collins et al. have recently demonstrated that the metabolic pathways we see disrupted by *Bmal1* deletion robustly cycle in synchronized macrophages (12). Overall, we are confident in the importance and relevance of our findings, and look forward to contextualization of our findings in wider physiological models.

**Figure 5:**
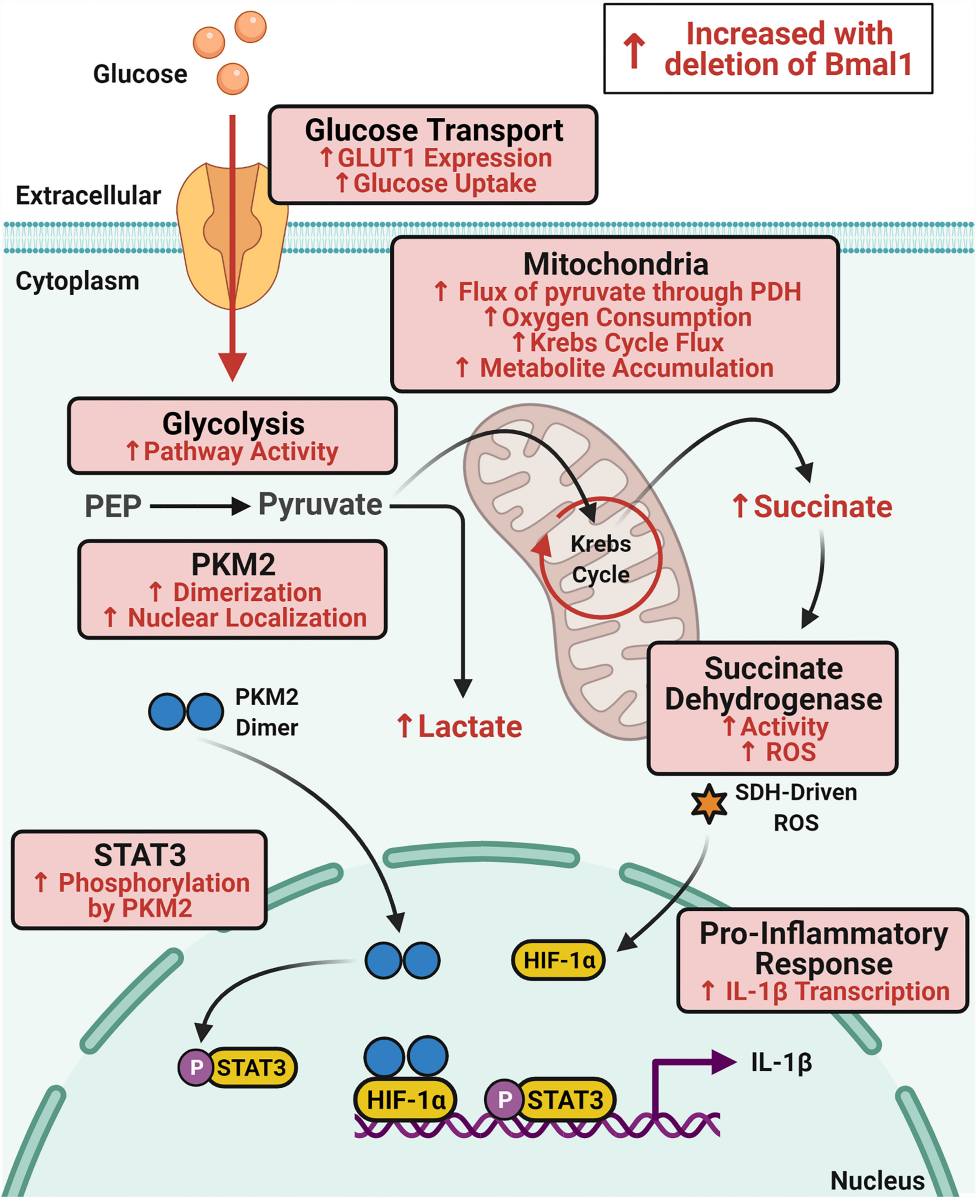
Schematic of immunometabolic changes in macrophages with deletion of Bmal1. Glucose metabolism is increased in macrophages with deletion of Bmal1 which potentiates expression of the pro-inflammatory cytokine IL-1β. In the absence of Bmal1 in macrophages, increased expression of the glucose transporter GLUT1 leads to increased glucose uptake and higher glycolytic pathway activity. Increased dimerization of the glycolytic enzyme PKM2 facilitates its translocation to the nucleus where it phosphorylates STAT3 to drive IL-1β expression. In the mitochondria, flux of pyruvate through pyruvate dehydrogenase (PDH) and oxygen consumption is increased alongside increased Krebs cycle flux which fuels accumulation of the intermediate succinate. Activity of the electron transport chain complex succinate dehydrogenase (SDH) is also increased in Bmal1*^-/-^* macrophages which produces heightened levels of ROS which stabilizes HIF-1α to also promote IL-1β expression. Therefore, BMAL1 is regulating glucose metabolism in macrophages to impact upon the expression of IL-1β.

In addition to immunity, metabolism is also extensively regulated by the molecular clock (80). The past decade of research has firmly placed immune cell metabolism as the critical regulator of immune cell fate and effector function (29). In parallel, studies have determined the key role of the molecular clock on immunity (8, 9). We are just beginning to integrate the three areas of the clock, immunity, and metabolism under the new area of circadian immunometabolism (40, 60, 78, 79). IL-1β is an archetypal pro-inflammatory cytokine and its expression is affected by metabolic alterations (26, 30, 39). Additionally, IL-1β is ubiquitous in the molecular pathogenesis of a chronic inflammatory diseases such as arthritis, cardiovascular disease, and autoimmune conditions (46), and clinical trials have demonstrated the potency of blocking IL-1β to ameliorate the molecular and systemic manifestations of these diseases (47–49). Our findings outline distinct metabolic pathways regulated by BMAL1 and link these changes to altered expression of IL-1β (**Figure 5**). Furthermore, these findings have clinical relevance. The expression of many existing drug targets is now known to be rhythmic (81) meaning that administrations of medicines can be timed to maximise efficacy and decrease side effects (82). This has been clearly demonstrated, for instance, by the use of timed glucocorticoid administration in early morning before the onset of peak symptoms in rheumatoid arthritis (83, 84). Therefore, targeting PKM2 or STAT3 at a discrete time of day may enhance efficacy, but this requires further investigation. In conclusion, our data illustrates a novel role of the circadian clock protein BMAL1 in macrophages as a metabolic sensor, which modulates levels of IL-1β through mitochondrial metabolism and immunometabolic control of the STAT3-PKM2 axis.

## Data Availability Statement

The raw data supporting the conclusions of this article will be made available by the authors, without undue reservation.

## Ethics Statement

The animal study was reviewed and approved by TCD Animal Research Ethics Committee (AREC) and RCSI Research Ethics Committee (REC).

## Author Contributions

GT and AC conceived project. GT, RC, EP-M, DF, EV, NJ, and AC designed research and critically evaluated results. GT, RC, JO’S, LF, SC, and MC-S performed experiments. GT, RC, MPC-S, EV, and NJ analysed data. GT and AC wrote the manuscript. RGC, EP-M, DF, EV, and NJ critically appraised the manuscript. All authors contributed to the article and approved the submitted version.

## Funding

This work was funded by an RCSI Strategic Academic Recruitment Program (StAR) award, a Science Foundation Ireland Career Development Award (17/CDA/4688) and an Irish Research Council Laureate Award (IRCLA/2017/110) all provided to AC.

## Conflict of Interest

The authors declare that the research was conducted in the absence of any commercial or financial relationships that could be construed as a potential conflict of interest.

## Publisher’s Note

All claims expressed in this article are solely those of the authors and do not necessarily represent those of their affiliated organizations, or those of the publisher, the editors and the reviewers. Any product that may be evaluated in this article, or claim that may be made by its manufacturer, is not guaranteed or endorsed by the publisher.
