# Supplementary material for: The Circadian Clock Protein BMAL1 Acts as a Metabolic Sensor In Macrophages to Control the Production of Pro IL-1β
Source: Front Immunol. 2021 Nov 9;12:700431. doi: 10.3389/fimmu.2021.700431 (PMC8630747; doi:10.3389/fimmu.2021.700431)
Supplement: Supplementary file 1 [file DataSheet_1.docx]

Supplementary Material

**
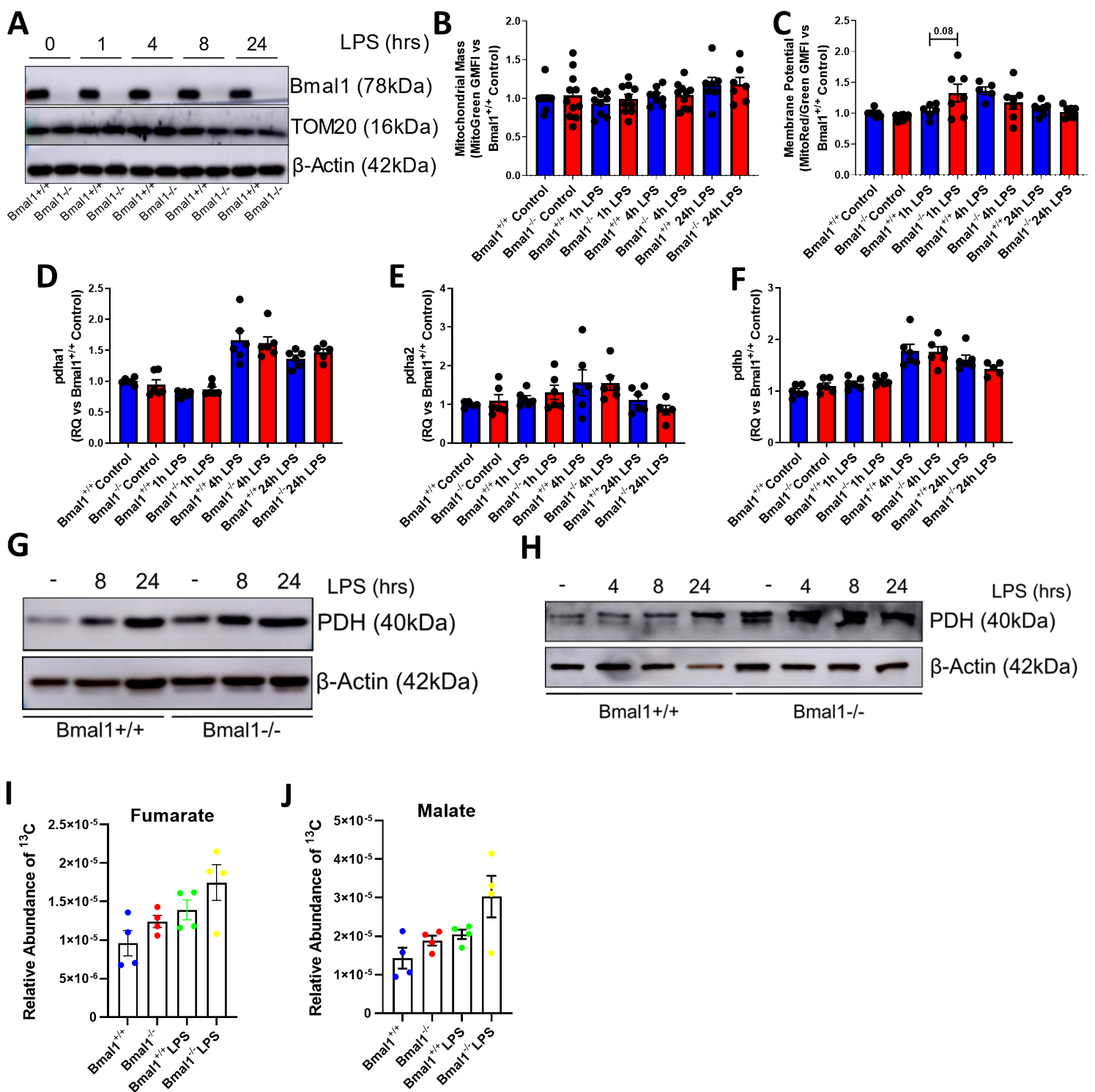
**

**Figure S1 – Mitochondrial respiration and Krebs cycle glucose flux is altered in Bmal1^-/-^ macrophages**

*Bmal1^+^*^/+^ and *Bmal1^-/-^* BMDMs were stimulated with LPS (100 ng/ml) and (A) lysed for Western blot analysis of TOM20 using β-Actin as a loading control. Data presented is representative of n=3 independent experiments. BMDMs were stained with (B) Mitotracker Green to measure mitochondrial mass and (C) Mitotracker Green and Red to measure mitochondrial membrane potential by flow cytometry. Data presented is at least n=4 +/- SEM. RNA was isolated and gene expression of (D) pdha1, (E) pdha2, and (F) pdhb was analysed by RT-qPCR. Samples were normalized to their expression of the housekeeping gene 18S. Data is presented relative to unstimulated *Bmal1^+^*^/+^ samples. Data presented is n=3 +/- SEM. (G, H) BMDMs were stimulated with LPS and protein expression of PDH was analyzed by Western blot using β-Actin as a loading control. *Bmal1^+^*^/+^ and *Bmal1*^-/-^ BMDMs were isolated, seeded in 10mM U-13C6 glucose, and stimulated with LPS for 8 hours. Cells were lysed and metabolites were quenched and measured via GC-MS to trace Krebs cycle flux of labelled glucose. Relative abundance of C-13 labelled (I) fumarate, and (J) malate was measured. Data presented is n=4 +/- SEM. Statistical analysis was performed for all data by one-way or two-way ANOVA with Sidak’s or Tukey’s multiple comparisons test (**p<0.01, ***p<0.001).

**
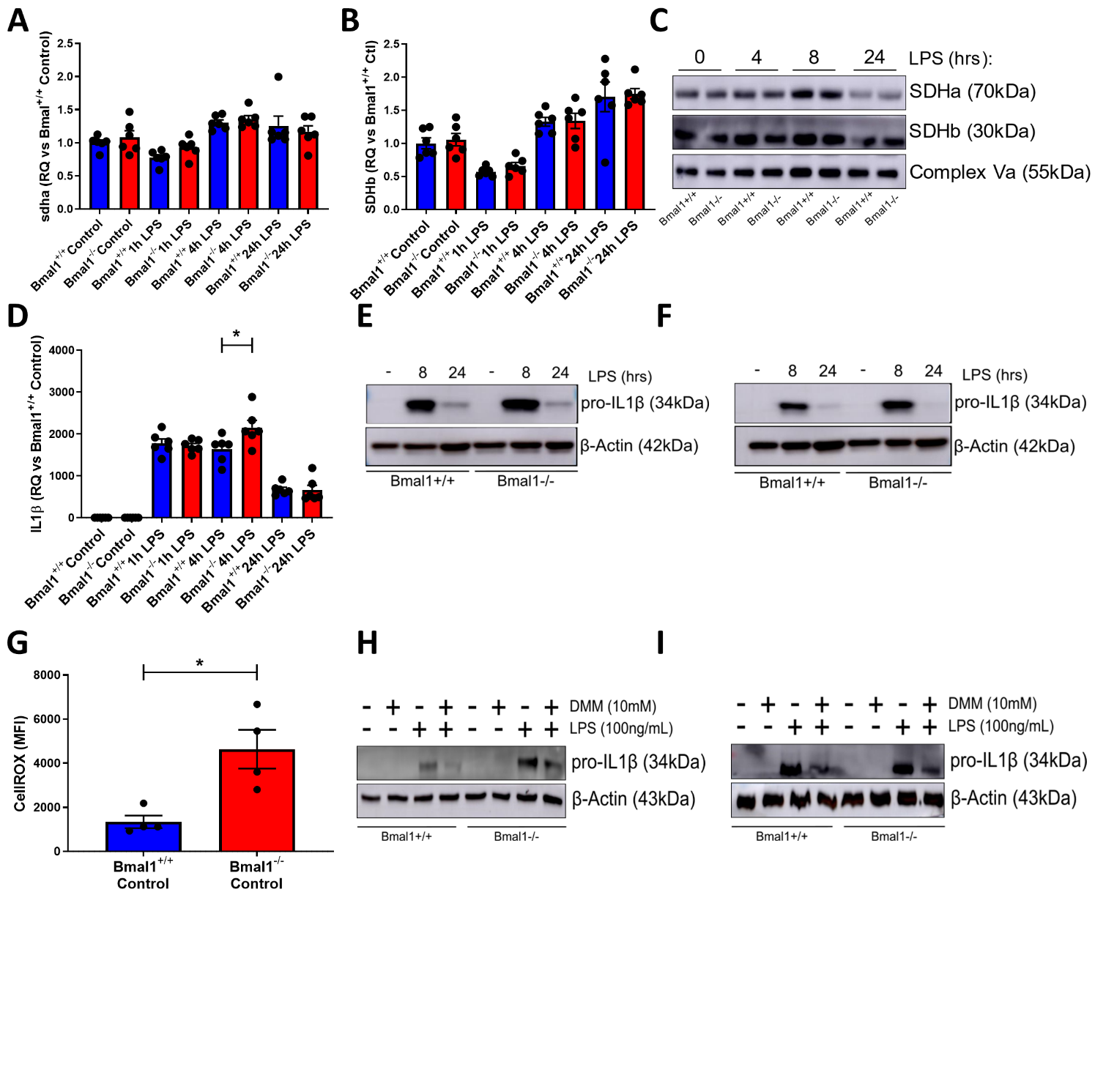
**

**Figure S2 – SDH-derived ROS promote increased IL-1β in macrophages with deletion of Bmal1**

*Bmal1^+/+^* and *Bmal1^-/-^* BMDMs were stimulated with LPS (100 ng/ml), RNA was isolated, and gene expression of (A) *Sdha*, (B) *Sdhb*, and (D) *Il1β* was analysed by RT-qPCR. Samples were normalized to their expression of the housekeeping gene 18S. Data is presented relative to unstimulated *Bmal1^+/+^* samples. Data presented is n=3 +/- SEM. Cells were also lysed for analysis of (C) SDHA and SDHB and (E, F) pro IL-1β protein expression by Western blot using Complex Va as a loading control and β-actin as respective loading controls. Pro IL-1β protein expression was also measured following pretreatment with DMM (H, I) before stimulation with LPS. (G) Cells from the peritoneal exudate of WT and *Bmal1^-/-^* mice were stained with α-CD11b and CellROX to measure, by flow cytometry, reactive oxygen species levels in peritoneal macrophages. Data presented is n=4 +/- SEM. Statistical analysis was performed for RNA data by one-way ANOVA with Sidak’s multiple comparisons test and for flow cytometry data by unpaired student’s t test (*p<0.05).

**
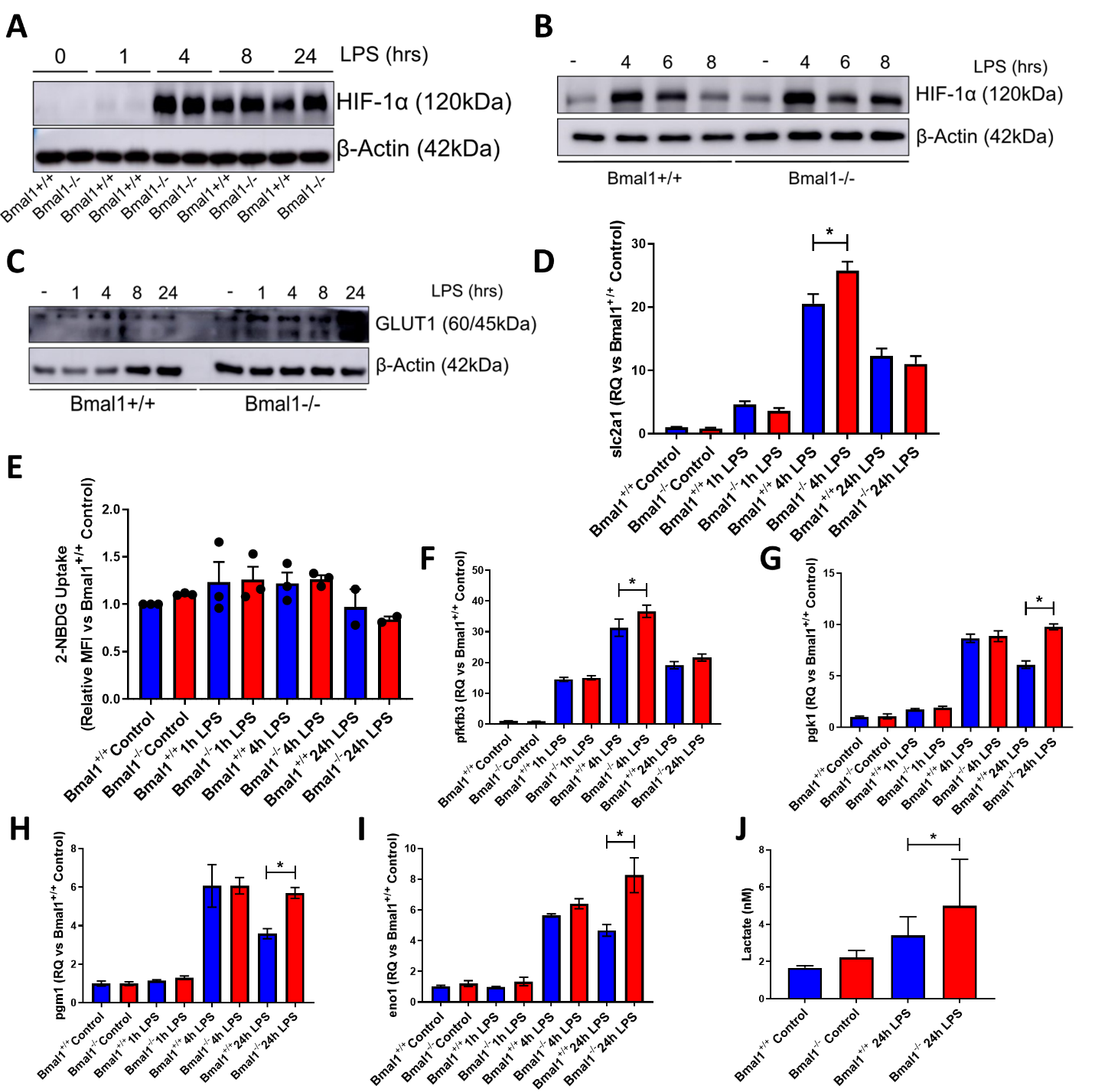

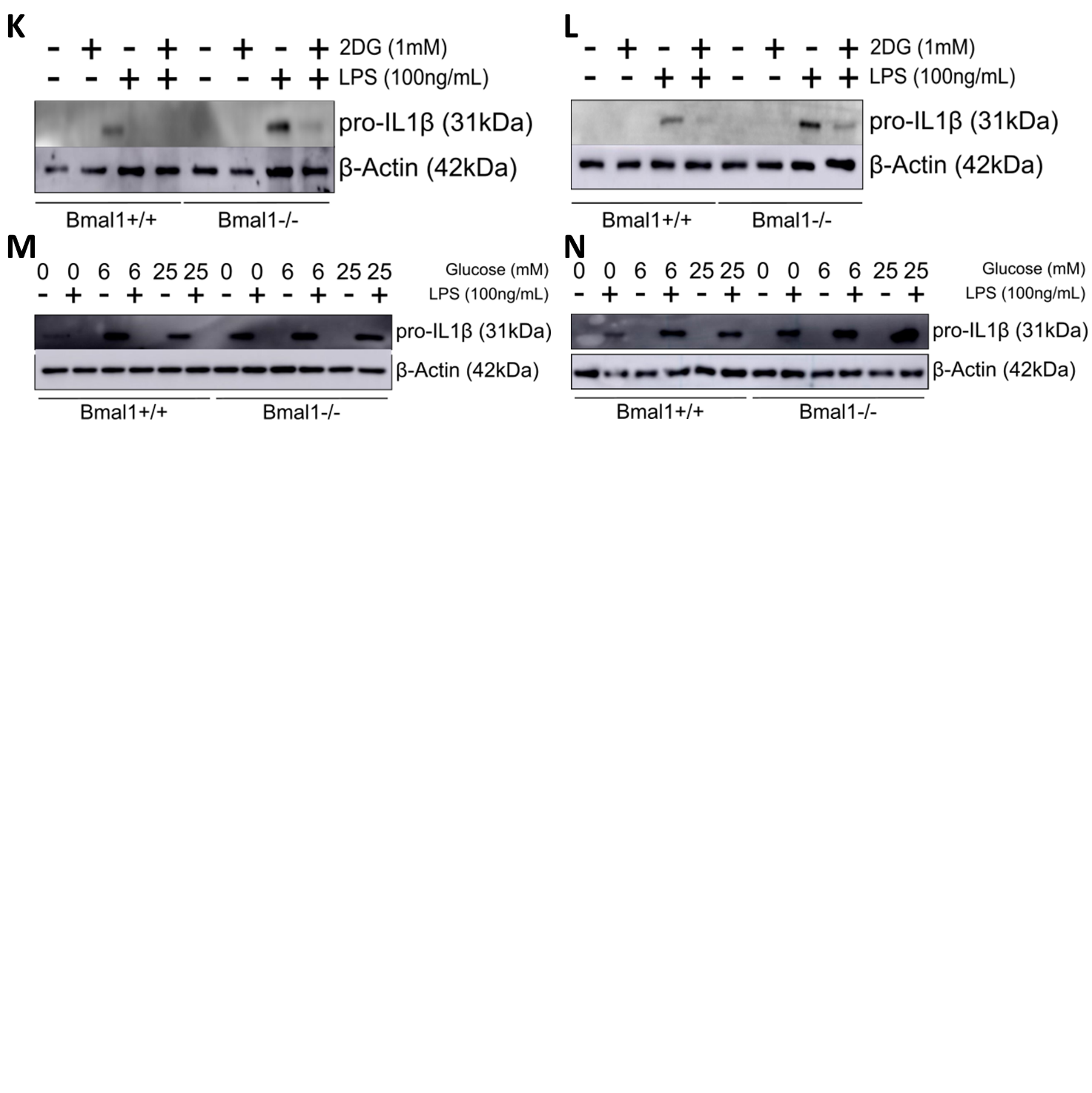
**

**Figure S3 – Glucose metabolism is upregulated and driving increased expression of IL-1β in macrophages with deletion of Bmal1**

*Bmal1^+/+^* and *Bmal1*^-/-^ BMDMs were stimulated with LPS (100 ng/ml). Protein expression of (A, B) HIF-1α and (C) GLUT1 was analysed using β-actin as loading controls. Protein expression of IL-1β was also analysed after (K, L) 2DG pretreatment and (M, N) glucose supplementation. RNA was isolated, and gene expression of (D) *Slc2a1*, (F) *Pfkfb3*, (G) *Pgk1*, (H) *Pgm1*, and (I) *Eno1* was analysed by RT-qPCR. Samples were normalized to their expression of the housekeeping gene 18S. Data is presented relative to unstimulated WT samples. Data presented is n=3 +/- SEM. (E) BMDMs were stimulated with LPS (100 ng/ml) and incubated with 2-NBDG to measure glucose uptake by flow cytometry. Data presented is n=3 +/- SEM. (J) BMDMs were lysed for analysis of lactate levels via luminescent assay. Lactate assay data presented is n=2. Statistical analysis was performed for all data by one-way ANOVA with Sidak’s multiple comparisons test (*p<0.05).


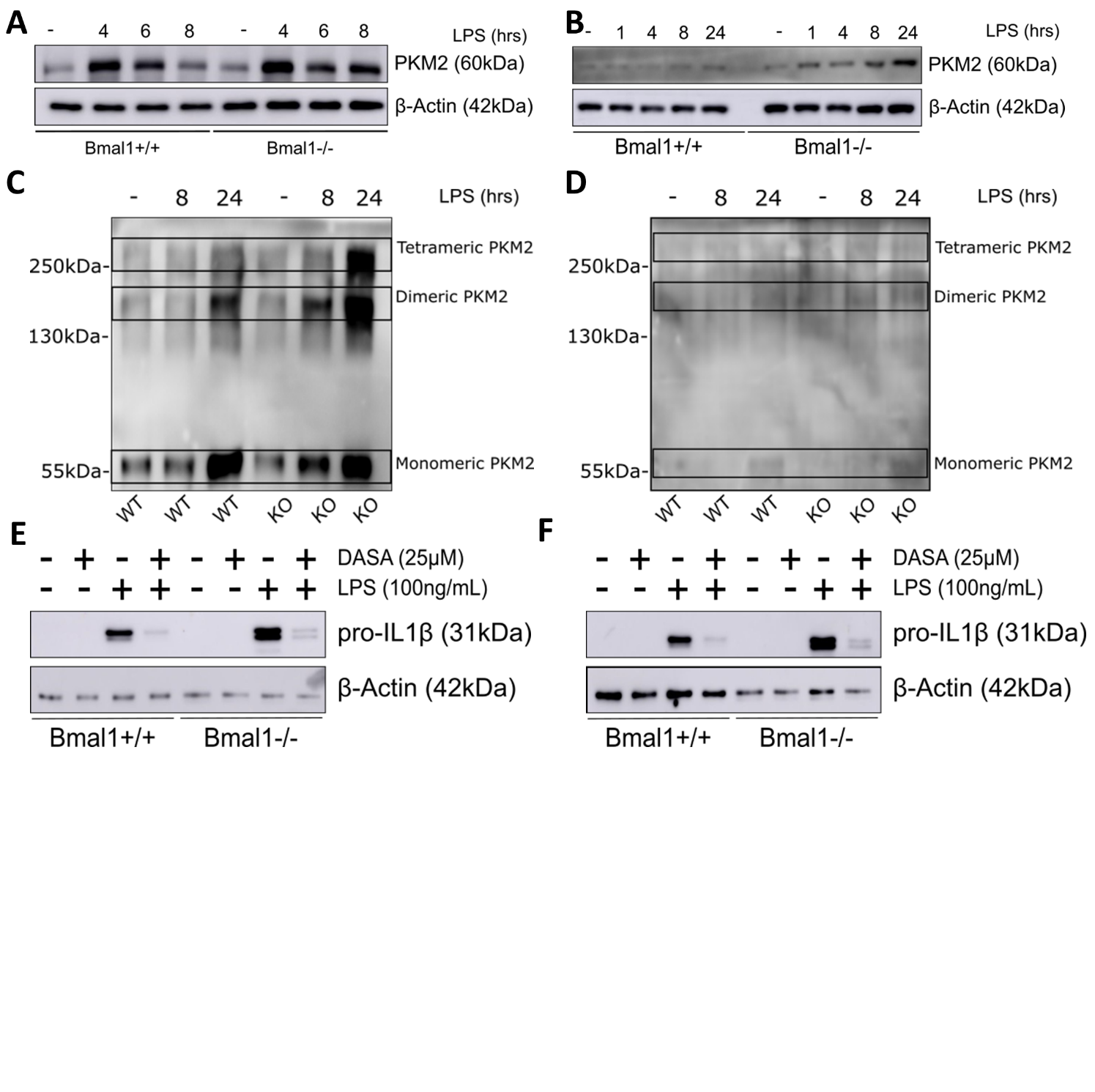


**Figure S4 – Nuclear PKM2 phosphorylation drive increased expression of IL-1β in Bmal1-/- macrophages**

*Bmal1^+/+^* and *Bmal1*^-/-^ BMDMs were stimulated with LPS (100 ng/ml) and protein expression of (A, B) PKM2 was analysed using β-Actin as a loading control. (C, D) PKM2 tetramers, dimers, and monomers were resolved by crosslinking samples after LPS stimulation before Western blot analysis. Pro IL-1β protein expression was measured following pretreatment with (E, F) DASA-58 (25 µM) before stimulation with LPS for 8 hours.

**
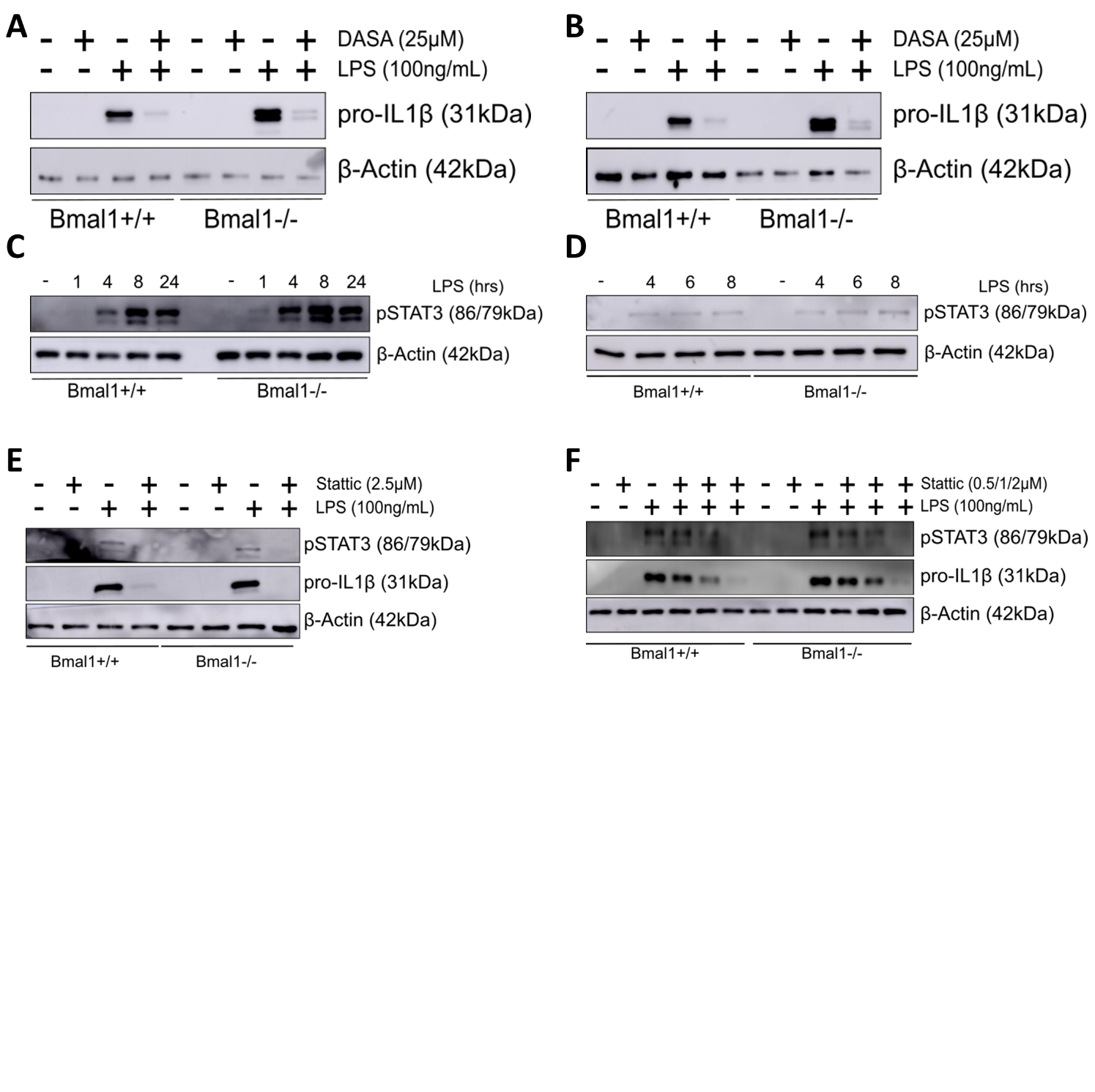
**

**Figure S5 – STAT3 phosphorylation drive increased expression of IL-1β in Bmal1-/- macrophages**

*Bmal1^+/+^* and *Bmal1*^-/-^ BMDMs were stimulated with LPS (100 ng/ml) and protein expression of (A, B) pSTAT3 was analysed using β-Actin as a loading control. (C, D) PKM2 tetramers, dimers, and monomers were resolved by crosslinking samples after LPS stimulation before Western blot analysis. Protein expression of Pro IL-1β was measured following pretreatment with (C, D) DASA-58 (25 µM) before stimulation with LPS for 8 hours. Protein expression of Pro IL-1β and pSTAT3 was measured following pretreatment with (G, H) STATTIC (0.5/1/2 µM) before stimulation with LPS for 8 hours. RNA was isolated, and gene expression of (E) IL6 and (F) TNFa was analysed by RT-qPCR. Samples were normalized to their expression of the housekeeping gene 18S. Data is presented relative to unstimulated WT samples. Data presented is n=3 +/- SEM. Statistical analysis was performed for all data by one-way ANOVA with Sidak’s multiple comparisons test (*p<0.05).
